# Mr.Bean: a comprehensive statistical and visualization application for modeling agricultural field trials data

**DOI:** 10.3389/fpls.2023.1290078

**Published:** 2024-01-03

**Authors:** Johan Aparicio, Salvador A. Gezan, Daniel Ariza-Suarez, Bodo Raatz, Santiago Diaz, Ana Heilman-Morales, Juan Lobaton

**Affiliations:** ^1^ Bean Program, Crops for Nutrition and Health, Alliance Bioversity-International Center for Tropical Agriculture (CIAT), Cali, Colombia; ^2^ Deparment of Statistical Genetics, InternationalVSN, Hemel Hempstead, United Kingdom; ^3^ Big Data Pipeline Unit, North Dakota State UniversityAES, Fargo, ND, United States

**Keywords:** spatial analysis, experimental designs, multi-environmental analysis, trial, breeding

## Abstract

Crop improvement efforts have exploited new methods for modeling spatial trends using the arrangement of the experimental units in the field. These methods have shown improvement in predicting the genetic potential of evaluated genotypes. However, the use of these tools may be limited by the exposure and accessibility to these products. In addition, these new methodologies often require plant scientists to be familiar with the programming environment used to implement them; constraints that limit data analysis efficiency for decision-making. These challenges have led to the development of Mr.Bean, an accessible and user-friendly tool with a comprehensive graphical visualization interface. The application integrates descriptive analysis, measures of dispersion and centralization, linear mixed model fitting, multi-environment trial analysis, factor analytic models, and genomic analysis. All these capabilities are designed to help plant breeders and scientist working with agricultural field trials make informed decisions more quickly. Mr.Bean is available for download at https://github.com/AparicioJohan/MrBeanApp.

## 1 Introduction

The selection of high-yielding and environmentally adapted genotypes in field trials is a fundamental challenge in plant breeding. In these types of trials, multiple genotypes are evaluated to estimate genetic parameters and determine the performance of traits of interest in breeding programs (Mackay et al., 2019). Experimental field design plays a crucial role in plant breeding (Piepho et al., 2022). Two experimental designs are widely used in traditional breeding field trials: (i) randomized complete block design (RCBD) and (ii) incomplete block design (Alvarado et al., 2020).

Field trials are usually designed to account for spatial heterogeneity, traditionally controlled by blocking. Researchers divide replicates into blocks, as in the so-called incomplete block design. However, spatial variation in trials cannot be fully captured, and has been recognized as a major source of experimental error (Yan, 2021). Spatial heterogeneity in the field can be associated with intrinsic biotic factors such as soil microorganisms, pests, diseases, and weeds. Abiotic factors also drive spatial heterogeneity, including the effects of soil fertility, nutrient concentration, presence of toxic elements, water availability, soil structure, and slope, among others. Agronomic management of the trial can also vary within and across sites (Isik et al., 2017). These conditions promote the generation of localized patterns or microenvironments that differ between experimental units in the field, reducing the overall uniformity of the trial (Bernardeli et al., 2021). For this reason, the experimental designs commonly used in plant breeding aim to separate genotypic information from the environmental variability (non-genetic variation). Separation of genotypic and environmental variability can improve selection accuracy in field trials, reducing the experimental error with increasing genetic gain (Cursi et al., 2021).

To model the genotypic and environmental components in a field trial, researchers use linear mixed models (LMM). These approaches contain a mixture of fixed and random effects to estimate and infer the variance components (Veturi et al., 2012). Some of these procedures can incorporate a component to model the spatial variation in breeding trials (Mao et al., 2020). Understanding spatial variation can improve predictions of the genetic potential of the evaluated genotypes. Towards this end, several approaches have been proposed to correct for spatial heterogeneity in the field (Cullis and Gleeson, 1991; Currie and Durban, 2002; Piepho and Williams, 2010; Robbins et al., 2012). There are two major classes of spatial analysis for field trials in plant breeding: (1) using neighboring plots to adjust the mean of the plot of interest, and (2) predicting the plot values by adding a spatial covariate to the mixed model (Zystro et al., 2018). These approaches can be further classified into those that use spatial variance-covariance structures and those using smoothing techniques to model spatial trends (Rodriguez-Alvarez et al., 2018).

One of the great challenges of in data analysis of plant breeding trials could is requires significant computational resources to process (Harrison and Caccamo, 2022). The complexity of the data and models can make it difficult these analyses. besides, the analysis of this data often involves multiple steps, including modeling, preprocessing, feature selection, and interpretation of results (Xu et al., 2022). Multiple software has been implemented with the aim of solving these problems. However, the implementation of these approaches into end-user tools is limited either by the accessibility of these tools or by the requirements and experience needed to program computer instructions for the models. Intending to help breeders or plant science researchers, this work describes “Mr.Bean”, a free R-Shiny application with a friendly and easy-to-use graphical user interface (GUI). This application simplifies the analysis of large-scale plant breeding experiments by using the power and versatility of LMM with or without spatial correction. This application combines the analytical robustness and speed offered by several R packages such as *ASReml-R* (Butler et al., 2017), *SpATS* (Rodriguez-Alvarez et al., 2018), and *lme4* (Bates et al., 2015) with the interactive features and visual power offered by Shiny R (Chang et al., 2023) and plotly (Sievert, 2020). The application also provides a graphical workflow for importing data from the Breeding Management System (BMS) and Breedbase through application programing interfaces (API), that help to identify outliers, and fit field data. Mr.Bean can analyze data from single-location or multi-environmental trials (MET), calculating the best linear unbiased estimator (BLUE), the best linear unbiased predictor (BLUP) (Piepho et al., 2008), and the broad-sense heritabilities. In addition, Mr. Bean offers a module for exploring results from Factor Analytic (FA) MET models using several graphical and multivariate techniques. The application integrates genomic and phenotypic data using the R-package *sommer* (Covarrubias-Pazaran, 2016). It estimates marker effects, variance components with genomic predictions, marker-base heritability, and genomic breeding values (GEBVs).

This application is a convenient and accurate way to analyze agronomic data, visualize field patterns and select genotypes for breeding programs. Mr.Bean aims to help statisticians, quantitative geneticists, and breeders who want to simplify and automate (or semi-automate) routine analysis to accurately predict the genetic potential of genotypes coming out of plant breeding pipelines. Moreover, Mr.Bean offers an alternative way to analyze field data for end-users with no previous experience in R programming language.

## 2 Methods

### 2.1 Mr.Bean implementation

Mr.Bean (v2.0.8) was developed in R using the package Shiny (Chang et al., 2023), an elegant and powerful web framework for creating R applications. Shiny supports developers with no previous experience using HTML, CSS, or JavaScript. Our developers improved the application’s interactive experience by employing additional extensions like ShinyJS, bs4dash, shinyWidgets, and ShinyBS. Mr.Bean uses a graphical interface designed to work under any web browser or R software as an R-Shiny application, executed in the x86_64-pc-linux-gnu (64-bit) platform. The core component consists of a set of 41 R attached packages, for r-base:4.1.1 or higher. Mr.Bean uses the packages *SpATS* (Rodriguez-Alvarez et al., 2018), *ASReml-R* (Butler et al., 2017), and *lme4* (Bates et al., 2015) for fitting LMM with or without spatial corrections. The *sommer* package (Covarrubias-Pazaran, 2016) within Mr.Bean integrates genomic information to estimate genomic best linear unbiased predictions (GBLUPs).

### 2.2 Running Mr.Bean

**Figure 1:**
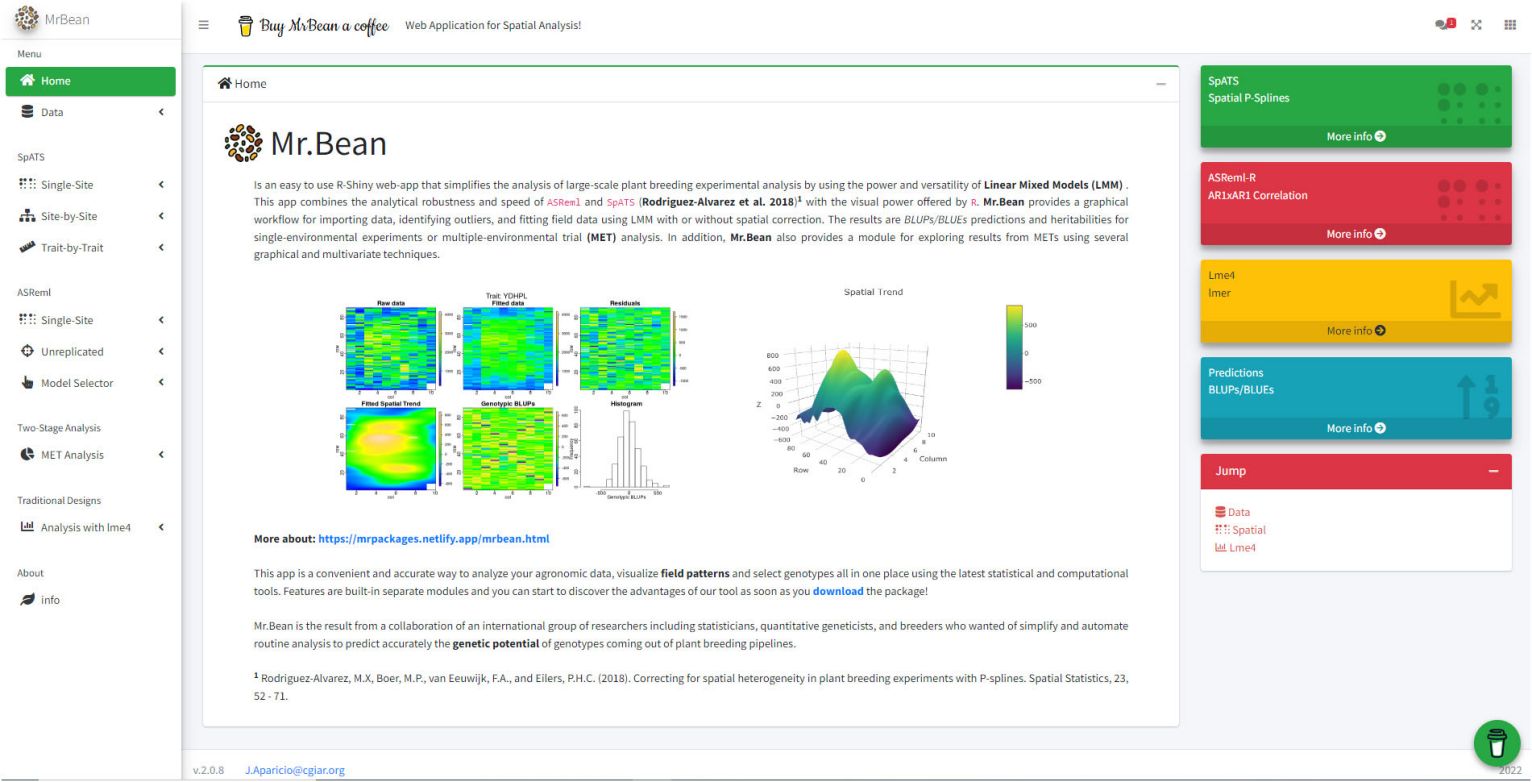
Mr.Bean application home page web.

**Figure 2:**
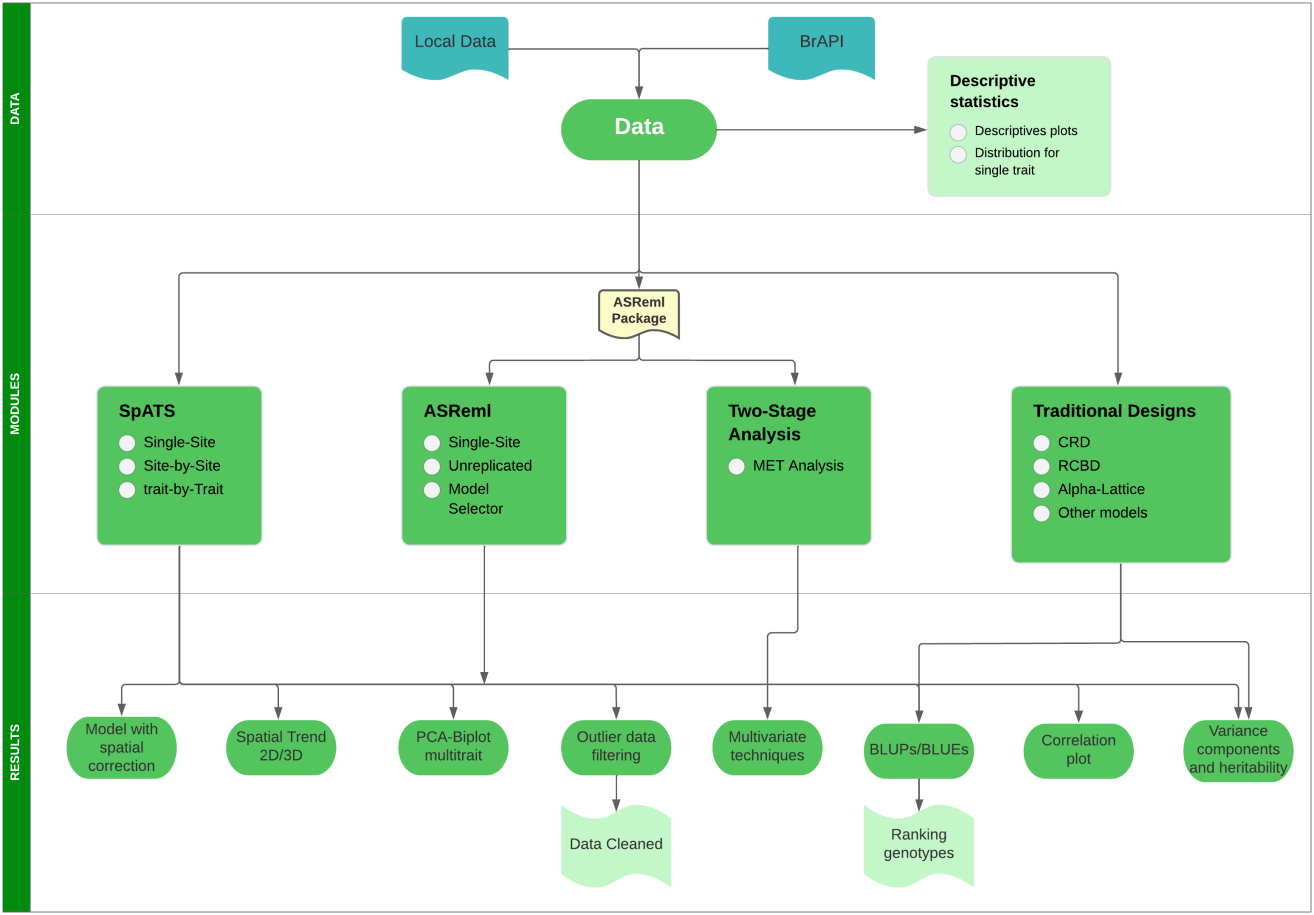
Flow diagram showing the logical process that Mr.Bean follows to perform several analyses.

Mr.Bean can be installed through the R software console from GitHub (https://github.com/AparicioJohan/MrBeanApp). It can also be installed and run locally by downloading it directly from the docker hub (https://hub.docker.com/r/johanstevenapa/mrbeanapp). For better understanding and ease in installing the application using GitHub or Docker, a video tutorial that explains the installation step by step is in the following link: https://www.youtube.com/watch?v=YubFj5DEQ2s. The application can be run in a beta version on the internet using any web browser for users without sufficient processing power, which anyone person can access through the following link: https://beanteam.shinyapps.io/MrBean_BETA/ (**Figure 1**). The beta version is a version that is hosted on a server of the Bioversity-CIAT alliance. The only disadvantage of this Beta version is that the *ASReml*, *Two-Stage analysis*, and *GBLUP* modules are not available and there must be a permanent internet connection. Mr.Bean follows a logical process through data loading, statistical analysis, model development, and results generation (**Figure 2**).

### 2.3 Data upload

The Data module allows users to upload their trial data. This module has several ways to import data from the Upload function. Data can be uploaded from your personal computer or via an internet connection to the Breeding (BrAPI) (https://brapi.org/), BMS and BreedBase APIs. The application is prepared to receive datasets with a maximum file size of 100 MB, following the tidy format in which every variable has a single column, and every observation a single assigned row (see Wickham, 2014 for a detailed explanation). Users can upload data in several formats, including comma-separated values (csv), tab-separated values (tsv), plain text (txt), and two different Excel formats (“xlsx” or “xls”). These upload capabilities allow users to identify the missing value character for their dataset.

Once the dataset has been uploaded, the module provides a quick view of the information for navigation (sorting, filtering, and pagination). Additionally, users can create subsets of variables for further analysis. The Descriptives section provides the ability to visually compare different qualitative and quantitative variables using box plots and two-dimensional scatter plots. The Distribution section helps visualize the frequency distribution for each individual trait using a histogram plot, with accompanying summary statistics such as mean, standard deviation, quartiles, and kurtosis, among others. For beginner users, a video tutorial on importing data and making plots in this section is available at the following link: https://www.youtube.com/watch?v=IlahWdDOOzU.

### 2.4 SpATS module

Here, the user can fit an LMM with spatial correction. SpATS is an attractive alternative to classical analyses of field trials, which model spatial variation as correlated noise (Rodriguez-Alvarez et al., 2018). It uses two-dimensional smoothing surfaces with penalized splines to model the spatial trends within the LMM framework. Hence, the implemented SpATS model is


$$y=\;\mu +gen+\;f_{u,v}\left(col,row\right)+\epsilon$$

where *y* is the trait of interest, *µ* is the overall mean, *gen* is the effect of the genotype, *f_u,v_(col,row)* are the row, column, bilinear polynomial, and smoothing spline effects, and *ϵ* is the effect of experimental error.

The *Single-Site* function allows the *SpATS* model to be run for experiments in a single location, evaluating one trait at a time. Users can calibrate the model with the *Model Specs* function. This function requires the user to specify the response variable, genotypes, and spatial coordinates for the plots, which are represented in rows and columns. At their discretion, users can select genotype checks for the trial and add additional variables as fixed or random effects, as well as covariates in the LMM. There is a *Help* button for beginner users that guides them step-by-step through each of the parameters required to run the model. The application generates a table with an estimate of the broad-sense heritability, residual standard deviation, R-squared, and coefficient of variation of the fitted model. Users can perform the Least Significant Difference (LSD) test if the genotype factor is selected as a fixed effect in the model. The application also produces tables and graphs summarizing the model’s variance components, spatial trends of raw data, fitted data, residuals, and genotype BLUPs with their respective histograms. Moreover, users can visualize spatial trends in the trial plots with two- and three-dimensional graphs.

The *BLUPs/BLUEs* subsection returns the predicted values for each genotype with their respective standard errors, including a histogram showing the distribution. The application also displays an error-bar plot that ranks the genotypic values for the variable of interest. Finally, the *Residuals* subsection provides tools to identify outlier observations from the analysis of residuals. It uses the assumption that residuals from the model follow a normal distribution with a mean of zero, using a 99% confidence interval to identify outlier data that fall beyond the range of ±3 standard deviations from the mean. The application graphs the outliers in field plots, identifying potential outliers or comparing residuals against other traits or factors. These functions contribute to the data cleaning process (quality assurance/quality check), before the user downloads a clean dataset.

The *Site-by-Site* function fits models for experiments evaluated in several locations, one trait at a time. This function also has a *Model Specs* subsection for fitting the model. As with the *Single-Site* function, the user selects the parameters required to run the model (response variable, genotype, spatial coordinates). The *Experiment* parameter allows the user to select sites for evaluation. Users can add other optional parameters (components with random or fixed effect, covariates). In addition, users can visualize the genotypes or lines that are shared between sites or experiments.

The *Results* subsection compares variance components between sites using a bar graph. As with the *Single-Site* function, the application summarizes spatial trends of raw data, fitted data, residuals, and genotype BLUPs with their respective histograms. The application creates a ranked error bar-plot of genotype BLUPs. Between evaluated experiments, the application generates correlation plots of phenotypic coefficients and their significance. Corresponding model components and summaries of each experiment are reported with the heritability estimated using the following equation:


$$H_{g}^{2}=\frac{ED_{g}}{m_{g}-1}$$

where ED_g_ is the effective dimension for genetic effects, and m_g_ is the number of genotypes (Rodriguez-Alvarez et al., 2018). As with other parts of this application, users can identify outliers and download clean datasets.

The *Trait-by-Trait* section has only one subsection, *Model Specs*. Users can run the model and observe the results for experiments evaluated at a single site, fitting multiple traits one at a time. This module was designed to compare the quantitative response of different variables. In plant breeding experiments, it is common to compare the behavior of one or more traits in one or more trials. This part of the application generates the same results described in the previous sections – spatial plots for each trait, summaries, model components, heritability, genotype ranking, outlier identification, etc. It also shows the genetics correlation between traits, offering a graphical display of Pearson’s second moment correlation coefficients, a dendrogram plot, and a Principal Component Analysis (PCA) for the traits and genotypes evaluated in the trial.

For beginner users, a video tutorial about *Single-Site*, *Site-by-Site*, and *Trait-by-Trait* analysis in this module is available at the following link: https://www.youtube.com/watch?v=QU_2O2ycZWA&t=303s.

### 2.5 *ASReml-R* module

Licensed researchers can use the *ASReml-R* and *Two-Stage-Analysis* modules. *ASReml-R* is a statistical software package for fitting linear mixed models using residual maximum likelihood (REML), as reported by Gilmour et al. (1995). The application for spatial analyses, establishes the natural variation in the data as the product of an autoregressive correlation (AR) structure for columns and rows denoted by AR1xAR1. *ASReml-R* is designed to fit the general LMM to moderately large datasets with complex variance models. The package has applications in the analysis of repeated measures data from multivariate analysis of variance and spline-type models, unbalanced design experiments, multi-environment trials, and regular or irregular spatial data (Butler et al., 2017). Many of these features are implemented in Mr.Bean.

Similar to the *SpATS* section, users can run the model for experiments in a single site using the *ASReml-R* function. Using the same interface as in previously described modules, the user selects the parameters of the response variable, genotype, and spatial coordinates with *Model Specs*. Optionally, users can include spatial coordinates (rows and columns) as splines or factors, and other covariates. The application generates spatial trend plots for raw data, fitted data, residuals, environmental variables, and genotype. It also generates a table with goodness-of-fit statistics, such as Akaike information criterion (AIC), Bayesian information criterion (BIC), heritability based on variance components (herit.VC), and heritability based on predictor error variance (herit.PEV), in addition to other statistics. Furthermore, the application generates a summary with the variance components, an ANOVA Wald test, and a 3D empirical variogram for the spatial trend of the residuals. In a *BLUPs/BLUEs* subsection, the *ASReml-R* module generates a table with predicted values and their respective standard errors and weights, a histogram of predicted values, and a ranking of genotypes using error bar plots.

In breeding trials, field experiments often test hundreds of genotypes with few or poor replications, mainly in the early stages of genotype screening. In these cases, checks are used to detect trends and allow the calculation of the residual variance. These trials using local controls assume that checks should have a similar response to the tested genotypes. Typically, augmented designs are the base for unreplicated trials, and their statistical analysis can be based on RCBD or on other spatial configurations (Gezan, 2023). For this reason, the *ASReml-R* module also allows fitting models for single-site unreplicated trials. The *Unreplicated* section presents a similar architecture to the *Single-Site* section by selecting the input parameters and the output results (spatial plots, residuals information, variance component, BLUPs, etc.). This section generates a table with goodness-of-fit statistics (AIC, BIC, herit.PEV, heritVC, A optimality, D optimality) to select the best spatial model by comparing the AR structure for columns, the AR structure for rows, or the AR structure for both spatial coordinates simultaneously.

The *ASReml-R* module can find the best spatial model for the data to be analyzed (*Model Selector* section). Similar to the other parts of this application, the user selects the available parameters. Mr.Bean then generates goodness-of-fit statistics. This section tests all the possible parameters for a model and then internally compares all the models to select the one with the best fit. Models are compared by block, complete blocks, splines, rows and columns, and the residual variance structures.

### 2.6 Two-stage analysis module

The *MET Analysis* function fits LMMs for multi-environmental trials using *ASReml-R*. This module has its own import data section, in a csv format, and it is independent from the other modules. Similar to the other modules, the user selects the parameters in the *Model Specs* subsection, providing the response variables, genotypes, and experiments, which are the different trials to be analyzed. The user will be able to analyze all trials of the dataset, selecting which trials to evaluate with the subset option. Additionally, there is an option allowing users to include weights in the two-stage analysis. These weights can be calculated by using the standard errors of the BLUEs, or by using the diagonal elements of the inverse of the variance covariance matrix associated with the genotype effect (Smith et al., 2001). In the option *Covariance structure*, the user can select the type of covariance structure to fit the model in the MET analysis. The list of the covariance structures being offered by Mr.Bean are diagonal (diag), uniform correlation (corv), uniform heterogeneous (corh), factor analytic 1 (FA1), factor analytic 2, (FA2), factor analytic 3 (FA3), factor analytic 4 (FA4), and US covariance matrix defined with correlations (corgh). The user can assess the data before running the model, by observing a barplot with the number of genotypes per trial, a heatmap for the shared genotypes between locations, and a barplot for means with standard errors for the selected trait.

The *Results* section shows a correlation matrix and dendrogram between trials evaluated. Also, a covariance matrix for trials is observed. Similar to the outputs of the previous modules, the application generates variance components, a summary of the model, residuals analysis, BLUPs for each genotype in each location, and a PCA biplot for the trials and genotypes (*GxE* option). Moreover, the section has a tool for comparing the model with different covariance structures using the likelihood ratio test (LR-statistic). When the factor analytic has been selected as a covariance matrix to fit the model, the *Factor analytic* section will be enabled. This section displays a bar chart for each factor selected, genotypic variance, and variance explained for each location. In addition, the latent regression can be reviewed for each of the genotypes in each of the selected factors. A dot plot with scores by genotype and a dot plot for loadings by environment is produced for each component selected.

### 2.7 Traditional designs module

Mr.Bean’s *Traditional Designs* module addresses the common lack of information about the spatial arrangement of field plots in trials. The module uses the R package *lme4* (Bates et al., 2015) to fit an LMM without spatial correction. The user must first select the response variable and genotype, before selecting the experimental design. In Mr.Bean have been implemented some traditional experimental designs for plant breeding, such as completely randomized designs (CRD), RCBD, row-column design and alpha-lattice design. Mr.Bean provides these models to analyze data from these designs:

$y_{ij}=\;\mu +gen_{i}+\epsilon_{ij}$ for CRD.

$y_{ijk}=\;\mu +gen_{i}+rep_{j}+\epsilon_{ijk}$ for RCBD

$y_{ijk}=\;\mu +gen_{i}+rep_{j}+\;row_{k}{\left(rep\right)}_{j}+\;col_{k}{\left(rep\right)}_{j}+\epsilon_{ijk}$ for row-column design

$y_{ijk}=\;\mu +gen_{i}+rep_{j}+block_{k}{\left(rep\right)}_{j}+\epsilon_{ijk}$ for alpha-lattice design.

Where *y* is the trait of interest, *µ* is the overall mean, *gen* is the effect of the genotype, *block* is the effect of the block, *rep* is the effect of the replication, *col* and *row* are the effects of the spatial location and *ϵ* is the effect of the experimental error. Mr.Bean also offers the ability to specify any other model formula using the *lme4* syntax, which is similar to the regular mathematical notation for specifying linear models (Bates et al., 2015).

Like the *SpATS* module, the application provides the significance of the fixed effects in the model using the F statistic, and reports variance components, likelihood-ratio test information, and the broad-sense heritability estimate (Cullis et al., 2006), together with some regularly used information for comparing different fitted models, such as AIC and BIC. The user can also make multiple comparisons when the genotype is taken as a fixed factor. Likewise, as in previously described modules, this module provides an analysis of residuals using a QQplot, a histogram, an analysis of outliers, as well as a list of ranked genotypes.

### 2.8 *GBLUP* module

The last module implemented in Mr.Bean is the *GBLUP* module. The app allows integrate genomic and phenotypic data with the aim of performing genomic prediction analysis using the R-package *sommer* (Covarrubias-Pazaran, 2016). In the *Genomic Prediction* section, the user only must import the phenotypic data and the genotypic data. The markers genotypic data must be in numerical format (-1, 0, 1), import the genetic map with the physical positions of the markers is also possible. In the same section, the users only have to select the phenotypic variables they want to analyze and the model can be executed. The current method available for this kind of analysis is GBLUP.

Mr.Bean estimates the variance components with genomic predictions, marker-base heritability, and GEBVs for each trait evaluated. Accuracy data and reliability, correlation plots between predicted and observed values of GBLUPs and the estimated squared-marker effect for each physical position similar to the Genome-wide association studies (GWAS) can be observed. Finally, in the *Results* section, the app shows the predictions plot with the fitted and predicted valued for each genotype.

### 2.9 Testing dataset

The dataset comes from a breeding population (*Vivero Equipo Frijol* or VEF population) of common bean (*Phaseolus vulgaris L.*) developed by the Andean bean breeding program of the Alliance Bioversity-CIAT (Keller et al., 2020). For the single-site analysis, a subset of 260 genotypes of the VEF population was planted in 2022 at the Alliance Bioversity-CIAT’s Palmira experimental field station (Colombia, 1,000 m a.s.l. altitude, latitude 3°32′N and longitude 76°18′W), under drought and irrigation.

For multi-environmental trial analysis, a historical dataset of 1,142 genotypes was planted at the Palmira experiment station, and at two additional sites: Darien, Colombia, with an altitude of 1,600 m a.s.l., (latitude 3°55′N and longitude 76°29′W) and Quilichao, Colombia, with an altitude of 1,000 m a.s.l. (latitude 3°1′N and longitude 76°28′W) over a period of seven years (2013, 2014, 2015, 2016, 2017, 2018, and 2019). For Darien, the trials were planted under three levels of phosphorus concentration – high phosphorus, medium phosphorus, and low phosphorus with optimal precipitation conditions (590 mm) for these trials. For Palmira, the trials were planted under drought and irrigated conditions. In Quilichao, the trials were planted under drought conditions. In total, 13 different trials were conducted (**Supplementary Table 1**).

The experimental units were row plots of 2.22 m^2^ laid out for each replicate of each genotype. The experimental design was an alpha-lattice with two and three replicates. Four traits were evaluated and reported in both datasets. The number of days to flowering (DF) was measured from the planting day to when 50% of the plants in the plot had at least one open flower. Days to physiological maturity (DPM) was measured as the number of days from planting until 50% of plants had at least one pod that had lost its green pigmentation. Yield (YDHA, kg ha^−1^) was determined for each plot and corrected for seed moisture of 14%. Seed weight (SW100, g 100 seeds^−1^) was obtained from 100 seeds (Diaz et al., 2020).

## 3 Results

### 3.1 Single site analysis

**Figure 3:**
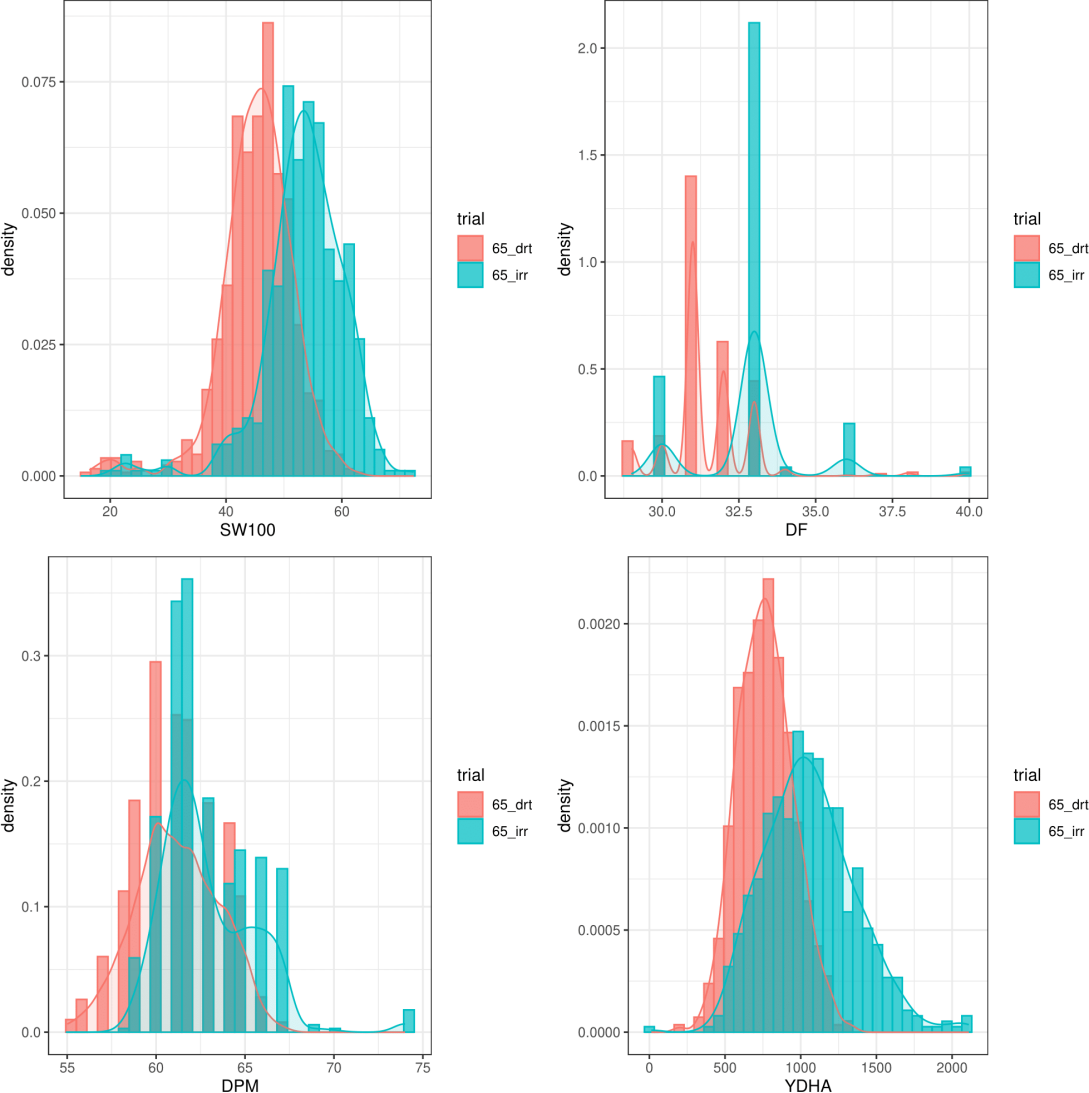
Phenotypic distribution of 100 seed weight (SW100), days to physiological maturity (DPM), days to flowering (DF) and yield (YDHA) of 260 lines belonging to VEF evaluated in drought (red plot) and irrigation (blue) conditions in 2022. (Figure generated directly by Mr.Bean).

**Figure 4:**
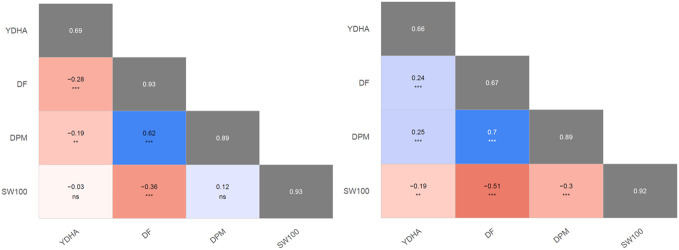
Pearson’s second moment correlation coefficients and their significances between best linear unbiased estimators (BLUEs) of evaluated traits. The broad-sense heritabilities of the best linear unbiased predictors (BLUPs) are located within the diagonal with the gray background. 100 seed weight (SW100), days to physiological maturity (DPM), days to flowering (DF), and yield (YDHA) of 260 lines belonging to VEF evaluated in drought (left side) and irrigation (right side) conditions in 2022. (Figure generated directly by Mr.Bean) Significance of correlations indicated as ***: p < .0001; **: p < .001; ns, not significant.

**Figure 5:**
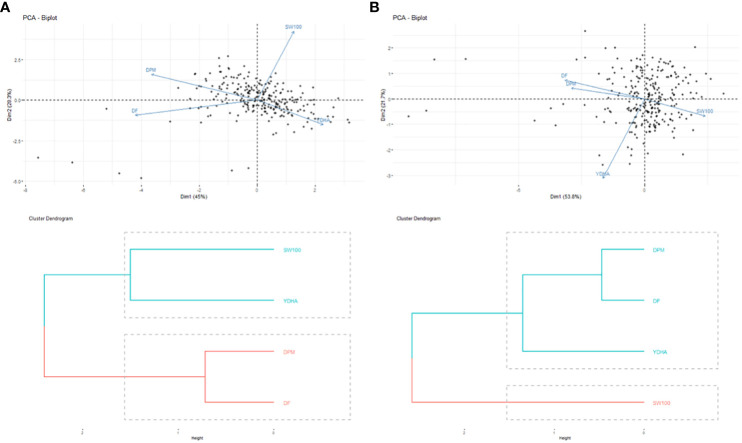
Biplot of principal components analysis (top side) and dendrograms (bottom side) of the phenotypic correlation for 100 seed weight (SW100), days to physiological maturity (DPM), days to flowering (DF) and yield (YDHA) of 260 lines (Black points) belonging to VEF population evaluated in: **(A)** drought (left side) and **(B)** irrigation (right side) conditions in 2022. (Figure generated directly by Mr.Bean).

Mr.Bean enabled the analysis of the phenotypic distribution of SW100, DPM, DF, and YDHA for 260 lines belonging to the VEF panel dataset, evaluated in Palmira under drought and irrigated conditions in 2022 (**Figure 3**; **Table 1**). Water availability conditions (drought and irrigated) affected SW100 and YDHA, two traits that also showed the highest coefficients of variation, 0.24 and 0.14 for drought and 0.29 and 0.13 for irrigation, respectively. The phenotypic correlation between the traits for the two conditions is shown in the correlation plot (**Figure 4**). In both conditions, a strong positive correlation was observed between DF and DPM (0.68 – 0.7). On the other hand, a negative correlation was observed between DF and SW100 (-0.35 – 0.5). YDHA was negatively correlated with DF and DPM under drought conditions. However, under irrigated conditions the correlation was positive. Mr.Bean generates a clustering dendrogram from the correlation matrix and a PCA biplot graph for the first two principal components of the distance matrix (**Figure 5**). The biplot shows the correlation between DF and DPM in both trial conditions (**Figures 5A, B**). **Figure 5** also shows the differences in the performance of the Mesoamerican genotype checks compared to the Andean genotypes.

**Table 1:** Summary statistics for phenotypic response of 100 seed weight (SW100), days to physiological maturity (DPM), days to flowering (DF) and yield (YDHA) of 260 lines belonging to VEF evaluated in drought and irrigation conditions in 2022.

	YDHA (kg ha^-1^)		DF		DPM		SW100 (g)	
	Dro	Irr	Dro	Irr	Dro	Irr	Dro	Irr
**Mean**	768.21	1057.64	31.51	32.89	61.18	62.87	45.31	53.48
**Std. Dev**	182.21	308.87	1.38	1.7	2.34	2.54	6.36	7.07
**Min**	191.83	12.82	29	30	55	58	16.4	20
**Median**	760.68	1040.48	31	33	61	62	45.6	54
**Max**	1341.17	2110.06	40	40	67	74	62.8	72.4
**CV**	0.24	0.29	0.04	0.05	0.04	1.15	0.14	0.13
**Skewness**	0.19	0.43	2.1	0.77	-0.03	1.15	-1.12	-1.31
**Kurtosis**	-0.09	0.43	10.47	3.59	-0.44	2.36	3.6	4.17

**Figure 6:**
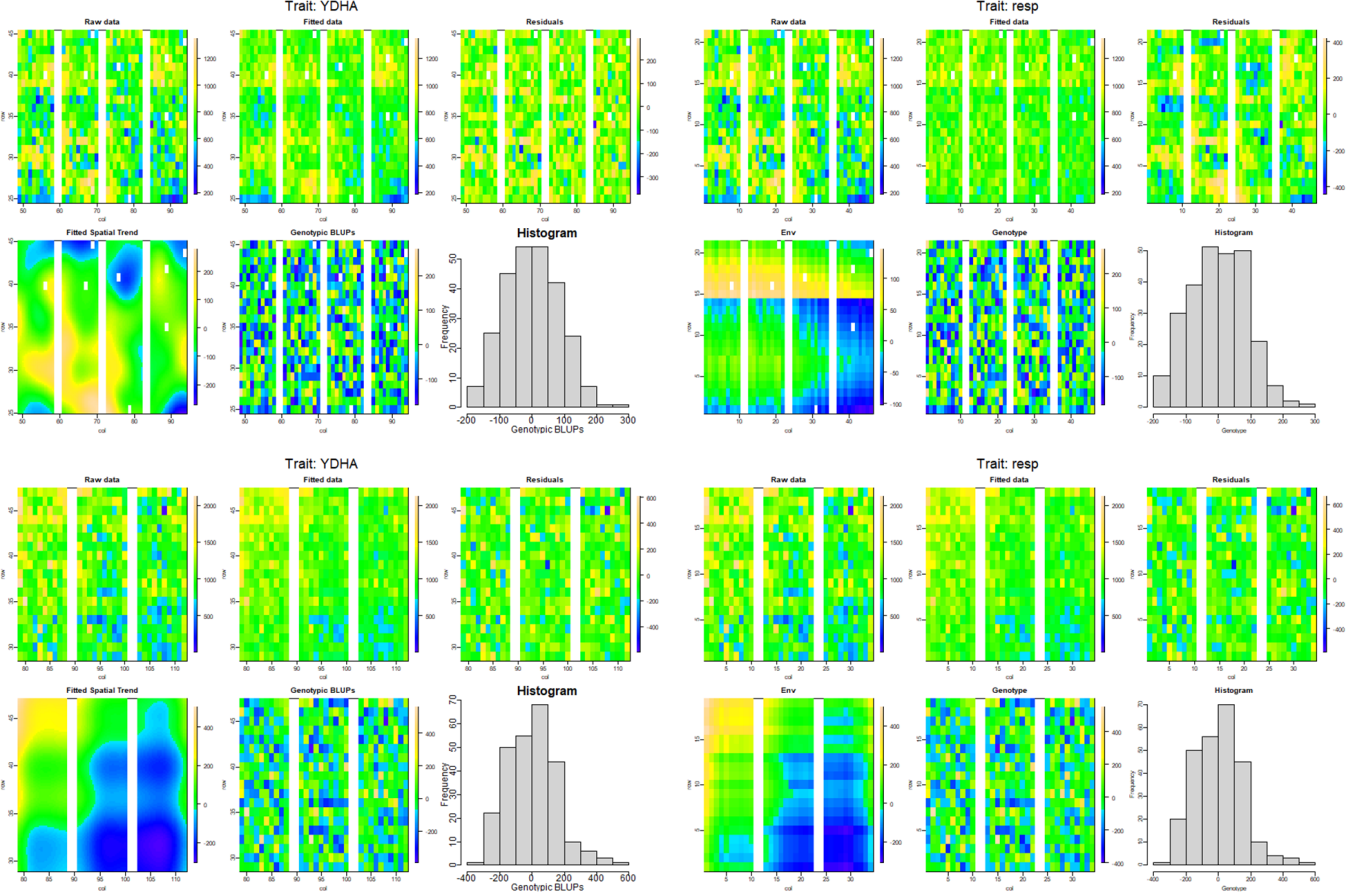
Spatial trends plots for raw data, fitted data, residuals, fitted spatial trend, and genotypic BLUPs for YDHA of 260 lines belonging to VEF population evaluated in drought (top side) and irrigation (bottom side) conditions in 2022. The models used for generating the spatial trends were SpATS (left side) and ASReml-R (right side) (Figure generated directly by Mr.Bean).

Model fitting was performed with *SpATS* (Rodriguez-Alvarez et al., 2018) and *ASReml-R* (Butler et al., 2017), using *lme4* under a row-column design (Bates et al., 2015) and considering the genotype effect as random. The heritability and variance components were then calculated. Next, the application calculated the spatial trends for raw data, fitted data, residuals, fitted spatial trend, and genotypic BLUPs for YDHA, using *SpATS* and *ASReml-R* models under drought and irrigated conditions (**Figure 6** and **Table 2**).

**Table 2:** Heritability and variance components for yield (YDHA), using *SpATS* (Rodriguez-Alvarez et al., 2018), *ASReml-R* (Butler et al., 2017), and row-columns design with *lme4* (Bates et al., 2015), of 260 lines belonging to the VEF panel dataset, evaluated under drought and irrigated conditions in 2022.

Model		Drought		Irrigation	
	Component	Variance	Std. Dev	Variance	Std. Dev
**SpATS**	**Genotype**	10260	101.3	34380	185.4
	**rep:col_f**	798.5	28.26	709.4	26.63
	**rep:row_f**	1104	33.23	1534	39.17
	**f(col)**	7813	88.39	17570	132.5
	**f(row)**	3302	57.46	57360	239.5
	**f(col):row**	651.5	25.52	17.63	4.199
	**col:f(row)**	0	0.001	0	0
	**f(col):f(row)**	59180	243.3	13.86	3.723
	**Residual**	11910	109.1	32500	180.3
	**Heritability**	0.69		0.66	
**ASReml-R**	**spline(row)**	809.769	1057.703	0.001	
	**spline(col)**	4128.431	4919.915	27206.11	21340.01
	**rep:row**	1003.913	585.48	4021.367	1801.49
	**rep:col**	2697.943	801.054	814.545	1134.622
	**Genotype**	10600.11	1499.37	33178.61	4723.381
	**row:col!col**	16005.85	1060.085	32862.8	2902.799
	**row:col!R**	1		1	
	**Heritability**	0.63		0.65	
**Row-Columns design with lme4**	**Genotype**	10621	103.06	32505	180.3
	**Rep:col_f**	3817	61.78	8336	91.3
	**Rep:row_f**	2024	44.99	12255	110.7
	**Residual**	16011	126.53	33871	184
	**Heritability**	0.63		0.62	

### 3.2 Multi-environmental trials analysis

**Figure 7:**
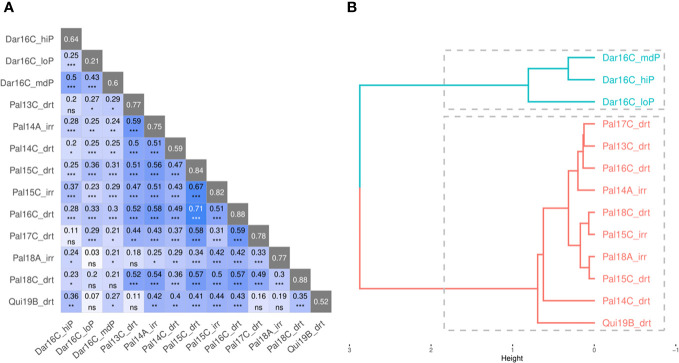
Correlation plot **(A)** and dendrogram **(B)** for yield (YDHA) of VEF population evaluated in 13 trials. (Figure generated directly by Mr.Bean). Significance of correlations indicated as ***: p < .0001; **: p < .001; *: p < .01; ns, not significant.

Mr.Bean evaluated the phenotypic distribution of SW100, DPM, DF, and YDHA of the VEF population in 13 trials (**Supplementary Figure 1**). Similar to a single-site analysis, **Figure 7** shows the results for YDHA. The phenotypic correlation for YDHA between trials is shown in the matrix and dendrogram. The trials established in Darien and Quilichao clustered around two representative groups for the 13 trials, in contrast to the trials planted in Palmira (except Pal18A_Irr) (**Figure 7B**).

Mr.Bean fit the model for MET analysis using *SpATS* and *ASReml-R*, with a two-factor analytic covariance matrix for YDHA. The variance components were then calculated (**Table 3**). A PCA biplot graph was generated for the first two principal components of the genotype distance matrix (**Supplementary Figure 2**). A factor analytic structure allowed the generation of scores for each genotype, loadings for each trial evaluated, and weights in the MET model (**Supplementary Figure 3**). The Mesoamerican check genotypes grouped around a higher positive score for the first component (**Supplementary Figure 3A**). Similarly, the Darien and Quilichao trials grouped around a higher score for the second component (**Supplementary Figure 3B**).

**Table 3:** Variance components for yield (YDHA), using *SpATS* (Rodriguez-Alvarez et al., 2018), and *ASReml-R* with one analytic factor as covariance matrix (Butler et al., 2017) of VEF population evaluated in 13 trials.

Experiment	SpATS		ASReml-R	
	varG	varE	varG	PVE (%)
**Dar16C_hiP**	53372.31	53845.07	52765.65	24.4
**Dar16C_loP**	10605.63	35326.7	36767.6	36.9
**Dar16C_mdP**	30384.25	38648.48	31722.67	31.5
**Pal13C_drt**	73510.5	57540.4	68551.71	90.1
**Pal14A_irr**	68039.77	60178.02	49812.17	97.8
**Pal14C_drt**	39602.25	48152.43	54009.83	70
**Pal15C_drt**	50911.35	25350.02	29782.21	100
**Pal15C_irr**	164304.2	94217.9	76122.49	100
**Pal16C_drt**	115512.9	43428.83	118722.9	89.6
**Pal17C_drt**	94995.45	65750.95	25156.68	100
**Pal18A_irr**	220896.3	165857.7	56923.65	100
**Pal18C_drt**	214225.1	79941.68	109404.3	96.4
**Qui19B_drt**	82026.66	120853.8	17325.5	100

## 4 Discussion

Mr.Bean offers robust analytical tools and visualizations for plant breeders and plant scientist across different disciplines. Mr.Bean was developed by the Bean breeding program from Alliance Bioversity-CIAT in collaboration with other researches from different institutions. Initially thorough to support to Bean breeding program is today a widely sued tool by breeding programs across the world. Some of the crops and breeding programs that have successfully used Mr.Bean include common bean, tropical forages, rice and cassava breeding programs from Alliance Bioversity-CIAT; also barley, spring wheat, soybeans, dry beans breeding programs and research extensions centers at NDSU and UM that used it to analyses data for agronomic research experiments. Evaluations can be focused not only on plant breeding, but can also be applied to research in plant pathology, entomology, physiology, and other fields. The application was developed as an open-source and accessible tool with an easy-to-use graphical interface. Researchers can run Mr.Bean with any web browser.

Mr.Bean was developed in the R language, but no programming experience is required. However, researchers using R can customize the open-source application with individual modifications to meet their needs and requirements. Mr.Bean’s individual modules are easy to understand and accessible to novice users. The workflow starts with downloading, cleaning, processing, and filtering the raw data for further analysis. The modules can be used for different analyses depending on the nature and purpose of the trials being evaluated. Users can generate graphs and tables with detailed information for future interpretation. Mr.Bean includes several visual tools such as real-time interactive statistical graphs developed in the R Shiny package. These tools support understanding and analyzing the behavior of the raw or processed data.

Mr.Bean models spatial variability – one of the major sources of error in field trials (Singh et al., 2003). The application uses linear mixed models with spatial components of field experiments implemented with *SpATS* and *ASReml-R* packages. The application accommodates traditional experimental designs lacking spatial information, such as randomized complete block designs or alpha-lattice designs, and separates genotypic variance from environmental variance. Ultimately, Mr.Bean facilitates data analysis towards improving genetic gain and making breeding programs more efficient (Covarrubias-Pazaran, 2020).

With single-site and multi-environment trial analysis, Mr.Bean enables breeders to make better use of their data and more robust decisions about genotype performance by calculating BLUEs and BLUPs for every trait and every location, within and across sites. The application estimates the selection response and provides breeders with critical tools to select the best performing genotypes. In addition, Mr.Bean can adjust any variable as a covariate to estimate its effect on the trial. The application allows multi-trait and genetic correlation analysis, allowing the development of a selection index for implementation in breeding programs. Supplementary materials and education videos can be found at github https://github.com/AparicioJohan/MrBeanApp and Youtube https://www.youtube.com/@ndsubigdatapipelineunit5201/.

## Data availability statement

The original contributions presented in the study are included in the article/**Supplementary Material.** Further inquiries can be directed to the corresponding author.

## Author contributions

JA: Conceptualization, Investigation, Methodology, Software, Supervision, Validation, Visualization, Writing – original draft. SG: Formal analysis, Methodology, Software, Writing – review & editing. DA-S: Conceptualization, Investigation, Software, Writing – review & editing. BR: Conceptualization, Funding acquisition, Project administration, Resources, Supervision, Writing – review & editing. SD: Data curation, Formal analysis, Supervision, Writing – original draft, Writing – review & editing. AH-M: Investigation, Methodology, Software, Validation, Writing – review & editing. JL: Funding acquisition, Project administration, Resources, Supervision, Writing – original draft, Writing – review & editing.

## Supplementary material

Supplementary Table 1 Combination of location, year and conditions which established VEF population in each trial.

Supplementary Figure 1 Phenotypic distribution of 100 seed weight (SW100), days to physiological maturity (DPM), days to flowering (DF) and yield (YDHA) of VEF population evaluated in 13 trials. (Figure generated directly by Mr.Bean).

Supplementary Figure 2 Biplot of the first two principal components of the correlation for yield (YDHA) of 1146 lines (Black points) belonging to the VEF population, evaluated in 13 trials (blue arrows) (Figure generated directly by Mr.Bean).

Supplementary Figure 3 Scores of 1,146 lines belonging to VEF population (a) and loading factor of 13 trials by Factor analytic (b) (Figure generated directly by Mr.Bean). The size and color of each individual point correspond to BLUE values for each environment or genotype. big size points and dark blue color correspond to environments or genotypes with higher BLUE values and small size points and yellow color correspond to environments or genotypes with lower BLUE values.
