# Supplementary material for: Mr.Bean: a comprehensive statistical and visualization application for modeling agricultural field trials data
Source: Front Plant Sci. 2024 Jan 3;14:1290078. doi: 10.3389/fpls.2023.1290078 (PMC10792065; doi:10.3389/fpls.2023.1290078)
Supplement: Supplementary Table 1 — Combination of location, year and conditions which established VEF population in each trial. [file Table_1.docx]

| **Year** | **Location** | **Condition** | **Combination** | **Number of genotypes** |
| --- | --- | --- | --- | --- |
| 2013 | Palmira | Drought | Pal13C_drt | 216 |
| 2014 | Palmira | Irrigation | Pal14A_irr | 280 |
|  | Palmira | Drought | Pal14C_drt | 648 |
| 2015 | Palmira | Irrigation | Pal15C_irr | 380 |
|  | Palmira | Drought | Pal15C_drt | 380 |
| 2016 | Darien | Low phosphorus | Dar16C_loP | 385 |
|  | Darien | Medium phosphorus | Dar16C_mdP | 383 |
|  | Darien | High phosphorus | Dar16C_hiP | 380 |
|  | Palmira | Drought | Pal16C_drt | 381 |
| 2017 | Palmira | Drought | Pal17C_drt | 342 |
| 2018 | Palmira | Irrigation | Pal18A_irr | 379 |
|  | Palmira | Drought | Pal18C_drt | 326 |
| 2019 | Quilichao | Drought | Qui19B_drt | 299 |

**Supplementary Table 1:** Combination of location, year and conditions which established VEF population in each trial.
