# Supplementary figures and images for: Mr.Bean: a comprehensive statistical and visualization application for modeling agricultural field trials data
Source: Front Plant Sci. 2024 Jan 3;14:1290078. doi: 10.3389/fpls.2023.1290078 (PMC10792065; doi:10.3389/fpls.2023.1290078)

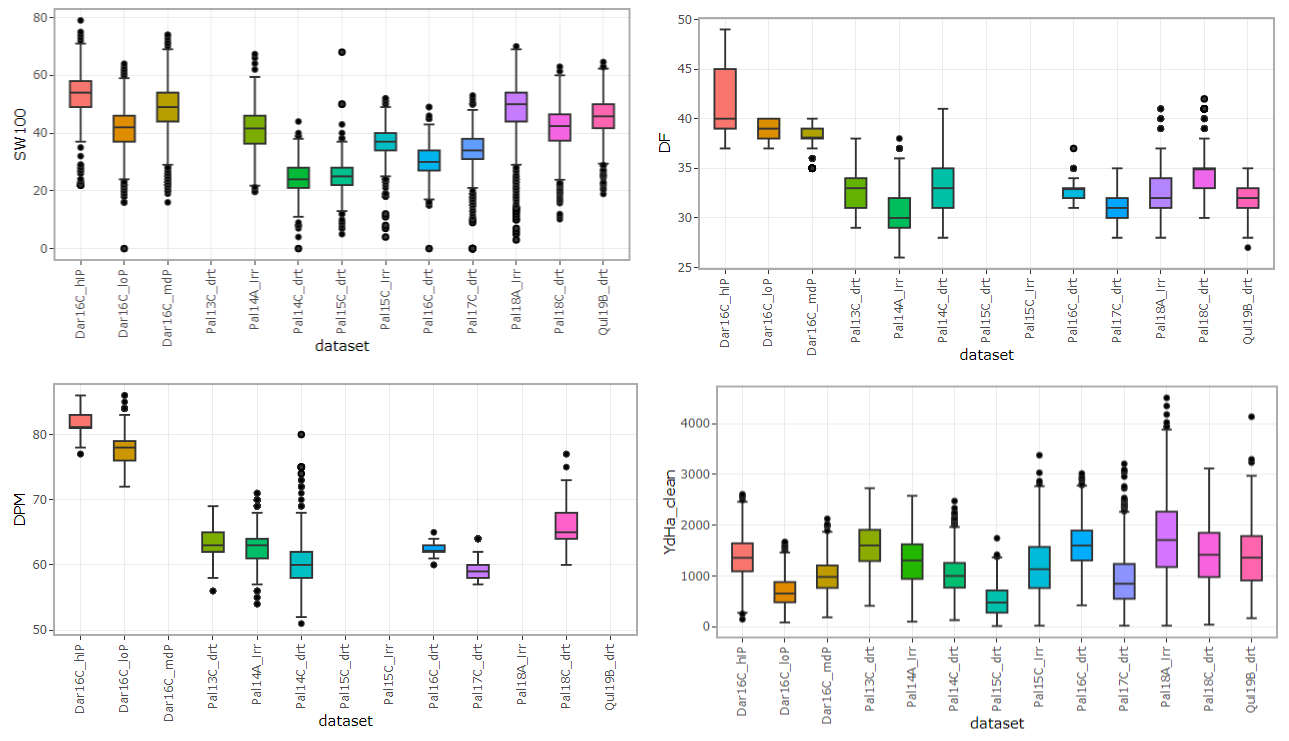

Supplement: Supplementary Figure 1 — Phenotypic distribution of 100 seed weight (SW100), days to physiological maturity (DPM), days to flowering (DF) and yield (YDHA) of VEF population evaluated in 13 trials. (Figure generated directly by Mr.Bean). [file Image_1.tif]

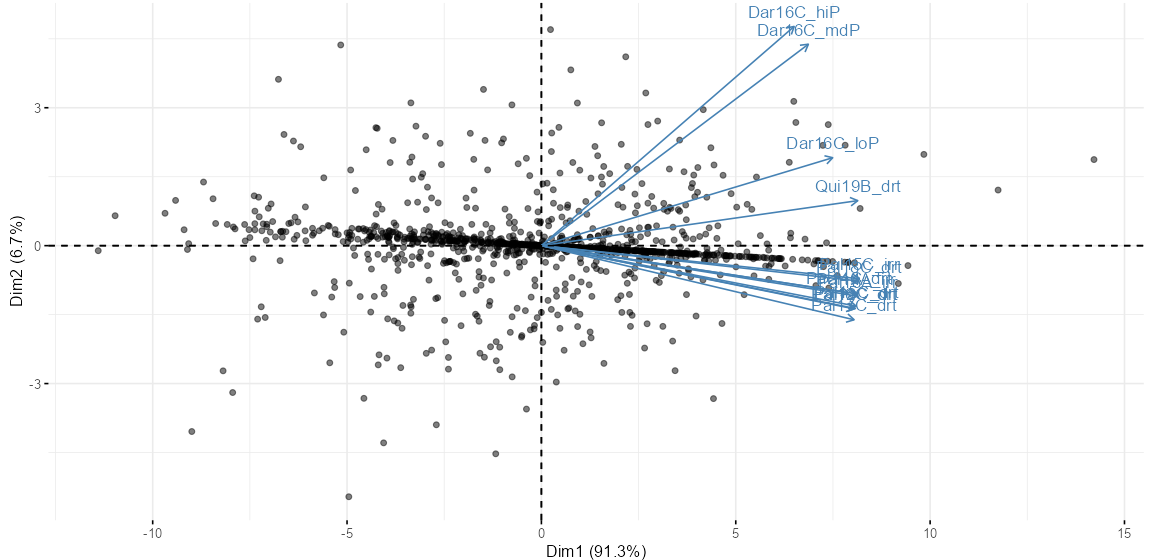

Supplement: Supplementary Figure 2 — Biplot of the first two principal components of the correlation for yield (YDHA) of 1146 lines (Black points) belonging to the VEF population, evaluated in 13 trials (blue arrows) (Figure generated directly by Mr.Bean). [file Image_2.tif]

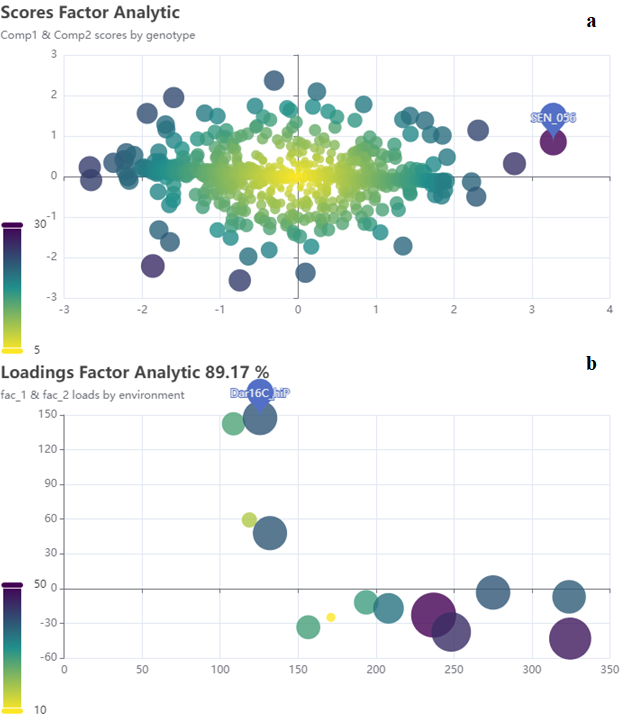

Supplement: Supplementary Figure 3 — Scores of 1,146 lines belonging to VEF population (a) and loading factor of 13 trials by Factor analytic (b) (Figure generated directly by Mr.Bean). The size and color of each individual point correspond to BLUE values for each environment or genotype. big size points and dark blue color correspond to environments or genotypes with higher BLUE values and small size points and yellow color correspond to environments or genotypes with lower BLUE values. [file Image_3.tif]
